# Bispecific antibodies mimicking factor VIII in hemophilia A: converting innovation to an essential medicine

**DOI:** 10.1016/j.rpth.2023.100173

**Published:** 2023-05-10

**Authors:** Cedric Hermans, Glenn F. Pierce

**Affiliations:** 1 Division of Haematology, Haemostasis and Thrombosis Unit, Saint-Luc University Hospital, Université catholique de Louvain, Brussels, Belgium; 2 World Federation of Haemophilia, Montreal, Quebec, Canada

**Keywords:** bispecific antibody, essential medicine, factor VIII, hemophilia A, innovation, World Health Organization (WHO)

## Abstract

Preventive subcutaneous treatment of severe hemophilia A with bispecific antibodies that mimic the action of coagulation factor VIII (FVIII) is emerging as an effective alternative to replacement therapy with intravenous administration of FVIII concentrates, either derived from plasma or produced by biotechnology. Access to this innovative therapeutic approach for a growing number of patients worldwide increasingly appears to be a priority public health strategy. Inclusion of FVIII mimetic bispecific antibodies on the World Health Organization essential medicines list would contribute to health equity in lower-income countries.

## Essentials

• New hemophilia agents called bispecific antibodies mimicking factor VIII are important treatments.
• The new medicine emicizumab is one and is effective administered under the skin instead of intravenously.
• Wider access to these antibodies worldwide appears to be a public health priority.
• Granting of WHO essential medicine status would lead to more equity for low-income countries.

Hemophilia A is characterized by a complete or partial deficiency of blood clotting factor (F)VIII. The consequence is the occurrence of abnormal bleeding episodes. In patients with complete or near-complete FVIII deficiency, bleeding may occur spontaneously, mainly in the joints and muscles. These bleeds (sometimes a single episode) usually lead(s) to irreversible joint destruction, causing pain and disabling functional limitations, the main complications of hemophilia [1].

The classic treatment for hemophilia A is replacement therapy to compensate for FVIII deficiency [2]. It is based on regular and preventive (prophylactic) intravenous (i.v.) administrations of FVIII concentrates that are purified from plasma or produced by biotechnology [2]. Recent advances have facilitated access to a wide range of FVIII concentrates that are free of infectious risk, produced in large quantities, and more slowly eliminated from the blood (so-called extended half-life FVIII) following their modification by PEGylation or fusion to the Fc fragment of immunoglobulins [3].

However, many limitations of these treatments remain: the burden of venous access, which is particularly problematic in young infants and children and some older patients; the short half-life of the administered factors imposing frequent injections (1-2 times a week for FVIII with an extended half-life) [3,4]; the fluctuations in plasma concentrations (in the form of a peak of activity after administration, followed by a progressive decay); and their immunogenicity responsible for the development of neutralizing antibodies occurring in some patients, especially during the initiation of treatment in infants.

Some limitations of replacement therapies may be resolved by exploiting the cofactor properties of FVIII. The main function of the latter is to catalyze the activation of FX by FIXa [5]. This role can now be taken on by a recently developed bispecific antibody, emicizumab (Hemlibra) (F. Hoffmann–La Roche and Genentech, Inc). This FVIII mimetic antibody binds to FIXa and FX and allows activation of FX into FXa, mimicking the cofactor activity of FVIII and generating a thrombin burst [6]. The advantages linked to the nature of this treatment are numerous: subcutaneous administration, prolonged half-life, nonrecognition by anti-FVIII antibodies present in patients who have become immunized to FVIII, and very low immunogenicity. In practice, therapy with emicizumab allows infrequent subcutaneous injections (weekly or every 2-4 weeks). It provides stable hemostatic activity, even in patients who have developed neutralizing FVIII antibodies [7,8]. Although difficult to quantify, it is estimated that emicizumab maintains a hemostatic activity equivalent to ≃15 % FVIII, which corresponds to conversion from a severe or moderate phenotype to a mild phenotype.

The therapeutic role of emicizumab has been studied and demonstrated in a large program of HAVEN clinical trials involving children and adults with hemophilia A, with and without inhibitors, treated weekly or less frequently, ie, every 2 to 4 weeks. These studies demonstrated the hemostatic efficacy and safety of emicizumab, with low rates of breakthrough bleeding episodes [9]. Rare thrombotic complications were observed. These were seen in patients cotreated with emicizumab and activated prothrombin complex concentrates (activated vitamin K-dependent clotting factors), a highly thrombogenic circulating combination of emicizumab, and large amounts of its activated substrates (FXa and FIXa) [10,11]. Importantly, the community is continuing pharmacovigilance for any evidence of increased prothrombotic complications in patients treated with emicizumab [12]. Rare cases of thromboses, some in conjunction with eptacog alfa (activated) use, have been reported [13].

We propose that emicizumab should be considered as an essential medicine by the World Health Organization (WHO) to facilitate adoption in lower-income countries where it would be very useful. The WHO Model List of Essential Medicines was created in 1977 as an important step toward ensuring more equitable access across socioeconomic strata [14]. While it is not a substitute for essential medicines lists of individual countries, the WHO list, which is periodically updated, provides an important benchmark for medicines considered important for human health. New medicines can be reviewed periodically by WHO for inclusion if they satisfy criteria that contribute to human health. Countries are under no obligation to follow WHO recommendations, but the WHO endorsement sets the stage for within-country advocacy to add a new medicine to the in-country essential medicines list. With respect to hemophilia, FVIII and FIX (mentioned generically without brand and distinction between plasma-derived or recombinant), FIX complex (coagulations FII, VII, IX, X) concentrate, desmopressin, and tranexamic acid are on the 22nd list of WHO (2021) [15].

Emicizumab is currently registered and available in many countries [9]. Although priority is given to patients with inhibitors, emicizumab is now available and administered to many patients with severe and moderate hemophilia A without inhibitors. It is conservatively estimated that there are >19,000 patients worldwide treated with emicizumab [16]. For example, in the United States, more than one-third of all persons with hemophilia A of all severities use emicizumab. The major limitation in lower-income countries where it is licensed is cost, although some differential pricing is used by the manufacturer. Addition of emicizumab to the WHO essential medicines list would facilitate advocacy in countries for inclusion in country-specific essential medicines lists and set the stage for negotiating differential pricing commensurate with that country’s resources.

For all patients with a severe hemophilia phenotype, the treatment explicitly recommended by the World Federation of Hemophilia (WFH) Treatment Guidelines is prophylaxis, ie, the regular and preventive administration of a treatment to prevent spontaneous joint bleeds [2]. However, this is an ambitious goal and a rarely encountered aspiration in most countries. The first obstacle in lower resourced countries is certainly the lack or limited access to FVIII concentrates. Another obstacle is the burden and limitations of FVIII replacement therapy (i.v. administration, fluctuating concentrations, large interindividual variations, and immunogenicity). Frequently, patients need to return to the hospital for prophylaxis, reducing compliance. With limited supplies, prophylaxis with FVIII concentrate often is low dose, which reduces but does not stop breakthrough bleeding events. For patients from low-income countries, in particular, subcutaneous treatment with bispecific antibodies has several practical advantages over i.v. substitution: low interindividual and intraindividual variability with no treatment individualization required (only the frequency of injections can be individually determined), no need for biological monitoring in routine practice, hemostatic effect readily detectable by activated partial thromboplastin time measurement, very limited equipment required for administration, and easy education of patients and their families.

Even in well-resourced countries, and despite multiple initiatives, meeting the requirement that 100% of patients are on prophylaxis and have virtually no spontaneous joint bleeds is proving to be an elusive goal. In addition, even patients treated with full dose prophylaxis who have no clinically detectable bleeding episodes often develop ankle arthropathy, a sign of uncontrolled subclinical bleeding due to inadequately low FVIII trough levels [17,18]. It remains to be proven that subclinical bleeding will not occur with emicizumab, but the higher trough levels would suggest a better protection.

Although emicizumab is neither a cure nor a monotherapy, its multiple properties make it a first-line treatment for almost all patients, potentially including newborns [19], with hemophilia A and a severe bleeding phenotype. For the first time, a treatment has been developed that allows effective prophylaxis in lower-income countries where FVIII prophylaxis was previously very challenging or only available to a minority of patients. More than 1000 patients in resource-limited countries (<1% of all candidates) currently have access to emicizumab through the WFH humanitarian aid donation program [20]. Some countries have included emicizumab in their pharmaceutical procurement, such as Côte d'Ivoire, where emicizumab is available and financially supported by the health authorities. It was recently demonstrated that emicizumab was instrumental in successfully implementing uninterrupted, highly efficacious, and well-tolerated prophylaxis among 33 Ivorian boys aged 2 to 13 years with and without inhibitors. Twelve months after initiating emicizumab, a 99% reduction in bleeding rates was observed, with a raise from 18% to 100% of boys having 0 spontaneous joint bleeds. Three boys required a single FVIII infusion following a traumatic bleed. Health-related quality of life measures significantly improved, and perception of treatment efficacy was positively rated in children and parents. Acceptance, tolerance, and adherence were excellent [21]. High target joint resolution and low breakthrough bleed rates consistent with the HAVEN studies were also recently reported among 433 patients with hemophilia from 22 sites in sub-Saharan Africa and provided with emicizumab through the WFH Humanitarian Aid Program [22].

In this context, emicizumab and other FVIII mimetic bispecific antibodies under development [23] should become a part of the WHO Essential Medicines Program as a class for the treatment of severe hemophilia A [15]. Priority should certainly be given to children and all patients with FVIII inhibitors to minimize joint damage and comorbidities, respectively. While in the coming years more effective therapies (such as gene therapy) may be on the horizon, the large unmet needs in lower-income countries are with us today.
